# Predictors of burnout, work engagement and nurse reported job outcomes and quality of care: a mixed method study

**DOI:** 10.1186/s12912-016-0200-4

**Published:** 2017-01-18

**Authors:** Peter Van Bogaert, Lieve Peremans, Danny Van Heusden, Martijn Verspuy, Veronika Kureckova, Zoë Van de Cruys, Erik Franck

**Affiliations:** 1 Nursing and Midwifery Sciences, Centre for Research and Innovation in Care (CRIC), Faculty of Medicine and Health Sciences, University of Antwerp, Universiteitsplein 1, B-2610 Wilrijk, Belgium; 2 Department of Nursing, Antwerp University Hospital, Wilrijkstraat 10, B- 2650 Edegem, Belgium; 3 Nursing and Midwifery Sciences, Centre for Research and Innovation in Care (CRIC), Department of Primary and Interdisciplinary Care, Faculty of Medicine and Health Sciences, University of Antwerp, Universiteitsplein 1, B-2610 Wilrijk, Belgium; 4 Mental Health and Wellbeing Research Group, Vrije Universiteit Brussel, Laarbeeklaan 103, 1090 Jette, Belgium; 5 Department of Health Care, Karel de Grote University College, Van Schoonbekestraat 143, B- 2018 Antwerp, Belgium

**Keywords:** Burnout, Work engagement, Job satisfaction, Turnover intentions, Quality of care, Structural equation model, Sensitizing concepts

## Abstract

**Background:**

High levels of work-related stress, burnout, job dissatisfaction, and poor health are common within the nursing profession. A comprehensive understanding of nurses’ psychosocial work environment is necessary to respond to complex patients’ needs. The aims of this study were threefold: (1) To retest and confirm two structural equation models exploring associations between practice environment and work characteristics as predictors of burnout (model 1) and engagement (model 2) as well as nurse-reported job outcome and quality of care; (2) To study staff nurses’ and nurse managers’ perceptions and experiences of staff nurses’ workload; (3) To explain and interpret the two models by using the qualitative study findings.

**Method:**

This mixed method study is based on an explanatory sequential study design. We first performed a cross-sectional survey design in two large acute care university hospitals. Secondly, we conducted individual semi-structured interviews with staff nurses and nurse managers assigned to medical or surgical units in one of the study hospitals. Study data was collected between September 2014 and June 2015. Finally, qualitative study results assisted in explaining and interpreting the findings of the two models.

**Results:**

The two models with burnout and engagement as mediating outcome variables fitted sufficiently to the data. Nurse-reported job outcomes and quality of care explained variances between 52 and 62%. Nurse management at the unit level and workload had a direct impact on outcome variables with explained variances between 23 and 36% and between 12 and 17%, respectively. Personal accomplishment and depersonalization had an explained variance on job outcomes of 23% and vigor of 20%. Burnout and engagement had a less relevant direct impact on quality of care (≤5%). The qualitative study revealed various themes such as organisation of daily practice and work conditions; interdisciplinary collaboration, communication and teamwork; staff nurse personal characteristics and competencies; patient centeredness, quality and patient safety. Respondents’ statements corresponded closely to the models’ associations.

**Conclusion:**

A deep understanding of various associations and impacts on studied outcome variables such as risk factors and protective factors was gained through the retested models and the interviews with the study participants. Besides the *softer* work characteristics — such as decision latitude, social capital and team cohesion — more insight and knowledge of the *hard* work characteristic workload is essential.

## Background

Thirty years of research on burnout and on nurse work environment provide a body of knowledge about occupational stress and well-being and insight in the psychosocial work environment of nurses, one of the largest workforce in healthcare. Both research domains started empirically with a lack of theoretical frameworks. Research on burnout and psychosocial work environment has predominantly been conducted using the *Maslach Burnout Inventory-Human Service Survey* [33]. The primary themes in burnout research fit readily into the *six areas of worklife* such as workload, control, community, fairness, reward and value congruence [31]. Engagement as the positive pole of a continuum and the opposite of burnout became an additional and interesting research domain to feature the person-job fit [41]. In line with Maslachs’ primary themes, Karasek and Theorell [25] have developed the job demand-control-support model that consists of three main dimensions: job demands, job decision latitude and job social support. This model provides insights about the mechanism of job related characteristics within specific nurse work environments such as emergency nursing, oncology nursing, mental health nursing and nurse unit managers [1, 2, 11, 16, 51]. Research on nurse work environment started with the observation that some hospitals in the US were more successful in attracting and retaining nurses compared to other hospitals. In addition, these researchers have been focused on to what extent certain relevant aspects were generalizable and transferable to other hospitals [34]. A substantial number of studies identified and linked aspects of a balanced, healthy and supportive psychosocial work environment ([20, 27, 30, 32]) with quality and patient safety indicators such as patient satisfaction, mortality, co-morbidity and adverse events [5, 6, 18]. Furthermore, intervention studies were conducted to evaluate quality improvement projects aiming practice environments that support highly motivated and skilled nurses answering accurately complex patient needs. In the US implementations of ANCC Magnet Hospital key components including transformational leadership, structural empowerment, exemplary professional practice and new knowledge, innovations and improvements [7, 56]. In the UK and other European countries implementations of or the Productive Ward – Releasing Time to Care™ program [35, 49, 54, 55].

Our research program was initiated more than 10 years ago, adapting these research insights and knowledge in the Belgian context and meanwhile aiming better understanding of the associations between nurse practice environment and nurse work characteristics such as workload, decision latitude and social capital and outcome variables such as feelings of burnout and engagement, nurse reported job outcomes and quality of care [42, 43, 45, 46, 50]. Our research initiatives have been contributing to a clear understanding of nurses their practice environment that could support and guide the practice community. Therefore, this study based on an explanatory sequential design, was a next step in a series of studies that developed comprehensive models providing a deep understanding of various associations and impacts on studied outcome variables. The study aims were threefold: (1) To retest and confirm two structural equation models exploring associations between practice environment and work characteristics as predictors of burnout (model 1) and engagement (model 2) as well as nurse-reported job outcome and quality of care; (2) To study staff nurses’ and nurse managers’ perceptions and experiences of staff nurses’ workload; (3) To explain and interpret the two models by using the qualitative study findings.

## Methods

This mixed method study was based on an explanatory sequential study design [15]. The study started in a first phase with a quantitative approach collecting and analysing of quantitative data with the aim to retest and confirm two previous developed models. The second phase, a qualitative study, existed of collecting and analysing qualitative data based on semi-structured interviews. Both study phases were conducted independently. Finally, in a third phase qualitative study results assisted in explaining and interpreting the findings of the two model.

### Study population

#### Quantitative data set

The study was conducted in two acute care university hospitals, one in the Dutch- and one in the French-speaking part of Belgium, with 600 and 850 beds respectively. All participants were staff nurses working in direct care in either medical, surgical, obstetric, geriatric or intensive care units and operating theatres including adult and paediatric care units. Participants were invited by one of the investigators to participate in the study on a voluntary basis. Data collection took place between September 2014 and May 2015. Respondents could complete the self-report questionnaires electronically either at home and/or in the hospital.

#### Qualitative data set

The purpose of the qualitative study was to investigate staff nurses’ and nurse managers’ perceptions and experiences of staff nurses’ workload. To understand the complexity of staff nurses workload we included for this study a purposive sample with typical cases of staff nurses as well as nurse managers practicing on medical or surgical units. Assuming that medical and surgical nursing units are relatively comparable in terms of staff nurse practice environment and nurse work characteristics such as *workload*, we might expect similar perceptions and experiences. Each staff nurse and nurse manager of the participating units were invited by two study investigators, respectively. Data were collected until sufficiency was obtained on the research topics (staff nurses = 9; nurse managers = 10). The semi-structured interviews were organized only in the Dutch-speaking university hospital between January 2015 and March 2015 and performed in a dedicated room. The hospital had recently implemented the Productive Ward programme and became involved in an accreditation process (JCI - Joint Commission International) as a part of a larger national hospital accountability process.

#### Ethical considerations

The institutional review board of each study hospital approved the qualitative study. In addition, a qualified ethics review committee (Antwerp University Hospital – University of Antwerp Belgium) approved the qualitative study.

### Procedure and data analyses

#### Quantitative study: model retesting and confirmation

The two models were carefully developed and fitted sufficiently to a cross-sectional dataset based on survey design. Moreover, we used a set of measurement instruments such as the *Revised Nursing Work Index* (NWI-R) [4], the *Maslach Burnout Inventory-Human Service Survey* (MBI-HSS) [33], the *Utrecht Work Engagement Scale* (UWES) [40], the *Intensity of Labour Scale* [38], *Social Capital* [17, 36] and *Nurse reported job outcomes* and *quality of care* [3, 42]. These measures were thoroughly tested with various study populations as well as in the present study regarding validity, reliability and consistency [42–48]. All measures used a 4-point Likert-type scale (strongly disagree, disagree, agree, strongly agree), where nurses were asked to rate their agreement, except for the MBI-HSS and UWES, where respondents rated frequencies on a 7-point scale ranging from never to every day.

**Fig. 1:**
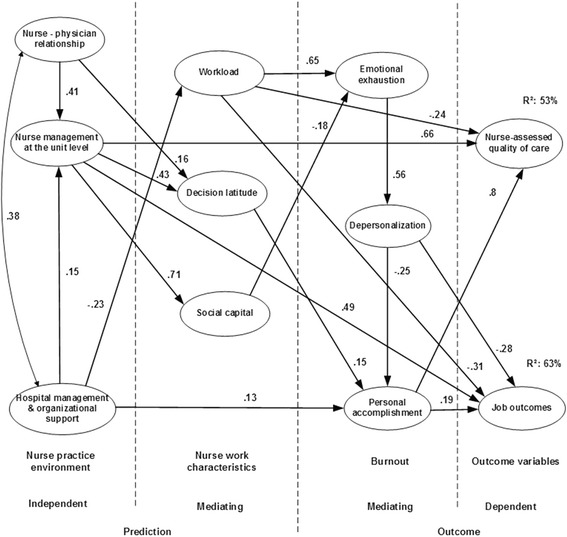
Model 1 - burnout as mediating outcome variable - retested model. Legend: All variables, with the exception of workload, emotional exhaustion and depersonalization were coded for analysis whereby higher scores indicated a stronger agreement or more favourable ratings. On the latter measures, higher scores are suggestive of unfavourable perceptions or conditions. All pathways were significant (p < .05). The independent variables of nurse practice environment predict the mediating variables of burnout dimensions, as well as job outcomes and nurse-assessed quality of care (dependent variables). In addition, workload, decision latitude, and social capital have a mediating position between the nurse practice environment and burnout dimensions. Nurse–physician relations and hospital management – organizational support impact nurse management at the unit level. Nurse management at the unit level has a strong direct impact on job outcomes and nurse-assessed quality of care as well as on decision latitude and social capital. Hospital management – organizational support has a direct impact on personal accomplishment and an indirect impact on the outcome variables through workload and burnout dimensions. Nurse–physician relations shows an indirect impact on the outcome variables through decision latitude. Social capital has an inverse impact on feelings of emotional exhaustion, and decision latitude supports feelings of personal accomplishments. Personal accomplishment, impacts indirectly by emotional exhaustion and directly by depersonalization, has a direct impact on job outcomes and nurse-assessed quality of care. The variances in job outcomes and nurse-assessed quality of care explained by this model were 63 and 53%, respectively. Nurse management at the unit level has a strong direct impact on outcome variables with explained variances of 25 and 36%, respectively

**Fig. 2:**
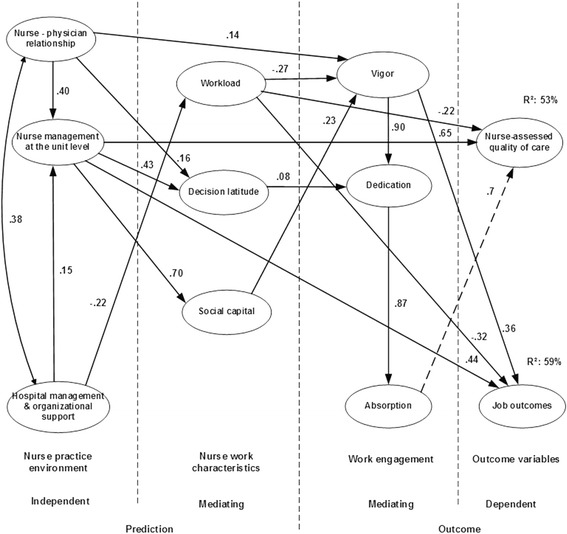
Model 2 – work engagement as mediating outcome variable – retested model. Legend: All variables, with the exception of workload, were coded for analysis whereby higher scores indicated a stronger agreement or more favourable ratings. On the latter measure, higher scores are suggestive of unfavourable perceptions or conditions. All pathways were significant (p < .05) except between absorption and nurse assessed quality of care (*p* = .076). The independent variables of nurse practice environment predict the mediating variables of work engagement dimensions, as well as job outcomes and nurse-assessed quality of care (dependent variables). In addition, workload, decision latitude, and social capital have a mediating position between the nurse practice environment and work engagement dimensions. Nurse–physician relations and hospital management – organizational support impact nurse management at the unit level. Nurse management at the unit level has a strong direct impact on job outcomes and nurse-assessed quality of care as well as on decision latitude and social capital. Hospital management – organizational support has an indirect impact on the outcome variables through workload and work engagement dimensions. Nurse–physician relations shows an indirect impact on the outcome variables through decision latitude. Social capital impacts feelings of vigor, and decision latitude supports feelings of dedication. Absorption, impacts indirectly by vigor and directly by dedication, has a direct impact on nurse-assessed quality of care. The variances in job outcomes and nurse-assessed quality of care explained by this model were 59 and 53%, respectively. Nurse management at the unit level has a strong direct impact on outcome variables with explained variances of 23 and 37%, respectively

These measures were used as variables to develop structural equation models describing associations between independent and mediating predictors such as practice environment and nurse work characteristics dimensions, respectively and mediating and dependent outcome variables such as burnout dimensions (model 1 see Fig. 1)/work engagement dimensions (model 2 see Fig. 2) and nurse-reported job outcomes/quality of care, respectively. In previous studies the population of the tested models included 1.201 staff nurses of two hospitals in the Dutch-speaking part of Belgium, and in one hospital group in the French-speaking part of Belgium [45, 50].

In SEM, a ratio of at least 5 subjects for each variable, including error measurements, observed variables (indicators) and latent variables (dimensions), is recommended [12]. A total of 85 and 80 variables (error measurements, observed and latent variables) were included in model 1 (burnout) and model 2 (work engagement) respectively and analysed in this study with a convenient sample of 751 respondents. Cronbach’s alpha coefficients of measures ranged from .639 to .913 (see Tables 4 and 5). However, job outcomes’ Cronbach’s alpha coefficient was in our studies low. Inter-item correlations, an alternative measurement technique assessing internal consistency [13], for the indicators of the job outcome dimension ranged from fair to moderate with values between .15 and .21 [45–48].

AMOS software was used to conduct model retesting and confirmation on the full database incorporating imputation of incomplete data, maximum likelihood estimation, and estimation of means and intercepts [8]. In our previous studies as well as in this study various fit measures were calculated and compared against accepted criterion levels (CFI and IFI ≥ .90; RMSEA < .080) to verify models plausibility.

The Statistical Package for the Social Science (SPSS) version 22.0 and AMOS version 22.0 software (SPSS Inc, Chicago) were used for descriptive analyses and computation of Cronbach’s alphas and correlation coefficients.

#### Qualitative study: semi-structured interviews

We used a descriptive phenomenological approach, from the staff nurse and nurse manager perspective about staff nurse perceived workload in daily practice. We aimed to reveal essential general meaning and structures about this phenomenon. Two investigators have performed individual semi-structured interviews with staff nurses and nurse managers, respectively. The interviewers use a topic guide starting from the *last personal experiences with perceived workload, aspects that influence perceived workload* and *impact of workload* (see Tables 1 and 2), which encouraged interviewer and respondent to go in-depth interaction. Each participant completed a short questionnaire about demographic characteristics. All interviews were audio recorded and study investigators took notes on non-verbal communication during the interviews. The two study investigators performed a descriptive thematic analysis with themes emerging from the data during the analysis. Researchers used also their field notes and put their own ideas carefully on paper before starting the analysis (bracketing). Credibility was achieved through the independent coding by two investigators, followed by comparing and discussing the codes and developing a codebook in consensus. The whole research team reflected on the results and discussed the rearrangement under the different themes [26]. Data collection and analysis occurred simultaneously; the codebook was developed iteratively, with the final codes confirmed before the final analysis was completed. The use of verbatim quotations ensured that the participants’ voices could be heard in the study [21, 37]. Moreover, as Sandelowski and Leemans [39] suggested each quote was clear separate reported in the results section. NVivo 10 software (QRS International) supported the qualitative thematic data analysis.

**Table 1:** Staff nurses’ semi-structured interview: topics and items

Topic	Items
Last experience with perceived workload	Describe the conditions and your actions?
	Could you handle the situation?
	What was the reaction of your team?
Aspects that influence perceived workload	What are the circumstances that you perceive workload?
	How do these circumstances occur? Do certain colleagues (nurses, physicians, physiotherapist, …) have a particular role in such a situation?
	In your opinion what is acceptable workload and what is not acceptable workload?
	Are there circumstances that you experience workload less fierce although there is lots to do? Why was that so?
Impact of workload	What is the impact of workload on yourself, physically and mentally?
	How do you deal after very busy workdays?
	Did you experience aversion to go to work caused by perceived workload?
	Do you have sometimes the intention to leave the nursing profession through your perceived workload?
	What is the impact of workload on your patients and on patient care

**Table 2:** Nurse managers’ semi-structured interview: topics and items

Topic	Items
Last experience with perceived workload	Describe the conditions?
	What was in your opinion the reasons that your staff nurses perceived workload? How did they cope?
	How did you have faced this situation and what were your particular actions?
Aspects that influence perceived workload	What are the circumstances when your staff nurses experience workload?
	How does these circumstances occur?
	In your opinion what is the impact of staff nurses’ competence, nurse - patient ratios and patient acuity on perceive workload?
	In your opinion what is acceptable workload and what is not acceptable workload?
	In your opinion can you and how do you adjust situations when your staff nurses perceive workload?
Impact of workload	What is the impact of workload on your staff nurses, physically and mentally?
	How do you deal with colleagues who experience difficulties with perceive workload?
	What is the impact on perceive workload on patients, patient care and safety?

#### Model analysis using the qualitative study findings

We performed a new analysis of the two models by using the qualitative findings. These findings could provide a deep understanding of the various associations and impacts on studied outcomes. The use of the qualitative data might have an additional value to strengthen models.

## Results

### Quantitative study: model retesting and confirmation

Response rate for each university hospital was 60% with a total sample of 751 participants (*n* = 425 and 326). Characteristics of study population and distribution of nurse reported job outcomes, nurse-reported quality of care as well as models’ observed and latent variables are summarized in Tables 3, 4 and 5, respectively. The two models (model 1 burnout and model 2 work engagement) fitted sufficiently to the data with CFI = 0.915 and 0.923, IFI = 0.916 and 0.924 and RMSEA = 0.041 and 0.043, respectively.

**Table 3:** Characteristics of study population and distribution of nurse reported job outcomes and nurse-reported quality of care (*n* = 751)

Nurse Characteristics	Mean	SD
Age in years	38.3	11.0
Years in nursing	14.6	11.3
Years on present unit	9.1	8.6
	N	%
Female	606	80.7
Baccalaureate degree in nursing or midwifery	611	81.3
Master degree in nursing and midwifery sciences	23	3.1
Working regime 50% or more of a full-time position	101	13.4
Working regime 75% or more of a full-time position	582	77.5
Outcome Variables	N	%
Dissatisfied or very dissatisfied with the current job	90	12
Intention to leave the current hospital within one year	44	5.9
Intention to leave nursing	69	9.2
The quality of care on the unit is fair or poor	107	13.2
The quality of care at the last shift is fair or poor	101	13.5
The quality of care in hospital the last year has deteriorated or definitely deteriorated	264	35.2

**Table 4:** Observed (a) and latent variables (b) of the retested models (*n* = 751)

Nurse practice environment:		loading model 1	loading model 2
Nurse-physician relationship (b) (Cronbach’s alpha: .83)			
2	Physicians and nurses have good working relationships (a).	.77	.77
27	Much teamwork between nurses and doctors (a).	.76	.76
39	Collaboration (joint practice) between nurses and physicians (a).	.87	.87
Nurse management at the unit level (b) (Cronbach’s alpha: .77			
33	Working with nurses who are clinically competent (a).	.54	.54
44	Nurse managers consult with staff on daily problems and procedures (a).	.45	.45
51	Standardized policies, procedures and ways of doing things (a).	.25	.25
Hospital management and organizational support (b) (Cronbach’s alpha: .83)			
14	A chief nursing officer is highly visible and accessible to staff (a).	.66	.66
36	An administration that listens and responds to employee concerns (a).	.82	.83
38	Staff nurses are involved in the internal governance of the hospital (e.g., practice and policy committees) (a).	.57	.57
Work characteristics			
Workload (b) (Cronbach’s alpha: .86)			
4	Many times I have to do a lot of work	.66	.67
7	Tasks that I have to solve are often very difficult	.85	.83
13	Normally time is short, so often I am pressed for time at work	.67	.69
Decision latitude (b) (Cronbach’s alpha: .68)			
2	To learn continuously is necessary in my work (a)^a^.	.33	.33
8	I can fully practice what I have learned in my training (a)^a^.	.69	.69
12	In my work I have to take a lot of decisions independently (a).	.29	.30
Social capital (b) (Cronbach’s alpha: .91)			
2	In our unit there is trust between nurses	.81	.81
4	In our unit there is favourable work climate	.77	.77
6	In our unit nurses shared values	.75	.75

^a^Superior fit indices were established by replacing two items of the decision latitude dimension

**Table 5:** Observed (a) and latent variables (b) of the retested model (*n* = 751)

Burnout:		Loading model 1	Loading model 2
Emotional exhaustion (b) (Cronbach’s alpha:. 90)			
1	I feel emotionally drained from my work (a).	.86	
2	I feel used up at the end of the workday (a).	.85	
14	I feel I’m working too hard on my job (a).	.67	
Depersonalisation (b) (Cronbach’s alpha:. 66)			
10	I’ve become more callous toward people since I took this job (a)	.51	
11	I worry that this job hardening me emotionally (a)	.73	
22	I feel patients blame me for some of their problems (a)	39	
Personal accomplishment (b) (Cronbach’s alpha: .69)			
17	I can easily create a relaxed atmosphere with my patients (a).	.60	
18	I feel exhilarated after working closely with my patients (a).	.85	
19	I have accomplished many worthwhile things in this job (a).	.67	
Work engagement:			
Vigor (b) (Cronbach’s alpha: .86)			
2	At my job, I feel strong and vigorous (a).		.82
5	When I get up in the morning, I feel like going to work (a).		.82
Dedication (b): (Cronbach’s alpha: .82)			
3	I am enthusiastic about my job (a).		.87
4	My job inspires me (a).		.73
Absorption (b) (Cronbach’s alpha:. 64)			
6	I feel happy when I am working intensely (a)^a^.		.72
9	I am immersed in my work (a).		.60
Outcome variables			
Job outcomes: (b) (Cronbach’s alpha: .32)^b^			
1	Job satisfaction (a).	.60	.64
2	Intention to stay in the hospital (a).	.39	.37
3	Intention to stay in nursing (a).	.28	.26
Nurse – assessed quality of care (b) (Cronbach’s alpha: .73)			
1	At the current unit (a).	.88	.88
2	At the last shift (a).	.77	.77
3	In the hospital the last year (a).	.49	.49

^a^Superior fit indices were established by replacing one item of the absorption dimension. ^b^Job outcomes’ Cronbach’s alpha coefficient was in our studies low. Inter-item correlations, an alternative measurement technique assessing internal consistency [13], for the indicators of the job outcome dimension ranged from fair to moderate with values between .15 and .21 [47]

Superior fit indices were established by replacing two items of the decision latitude dimension and one item of the absorption dimension. All pathways of the two models were significant except one pathway between absorption and quality of care (model 2) was not confirmed (*p* = .076). Nurse reported job outcomes and quality of care explained variances of model 1 (burnout) were 63 and 53% and of model 2 (work engagement) 59 and 53%, respectively. Hospital management/organizational support and nurse – physician relations had an indirect impact and nurse management at the unit level had a strong direct impact on outcome variables with explained variances of 25 and 36% in model 1 and 23 and 37% in model 2, respectively. Workload had an impact on outcome variables with explained variances of 15 and 13% in model 1 and 17 and 12% model 2, respectively. Personal accomplishment and depersonalization showed an explained variance on job outcomes of 23% and vigor of 20%. Personal accomplishment and absorption had less relevant direct impact on quality of care (≤5%).

### Qualitative study: semi-structured interviews

Staff nurses’ and nurse managers’ demographic characteristics are summarized in Table 6. The themes based on thematic analyses of the 9 staff nurses’ and 10 nurse managers’ interviews guided by the described topics were *organisation of daily practice and work conditions; interdisciplinary collaboration, communication and teamwork; staff nurse personal characteristics and competencies; patient centeredness, quality and patient safety.*

**Table 6:** Study population demographic characteristics qualitative study (*n* = 9; *n* = 10)

	Staff nurses	Nurse managers
	N	N
Total	9	10
Female	6	6
Age (years)		
20–30	3	2
31–40	1	2
41–50	5	1
51–60		5
Years in nursing		
<5	3	
5–10		
>10	6	
Years on present unit		
<5	4	
5–10	2	
>10	3	
Years as nure managers		
<5		2
5–10		3
>10		5
Diplome		
Baccalaureate degree in nursing	1	
Master degree in nursing	6	5
Additional management and leadership training		5
Working regime		
75%	7	
100%	2	10

#### Organisation of daily practice and work conditions

Perceived workload was not due to one factor but to a bundle of factors. These factors in staff nurses’ daily practice determined their workload. They have noticed increased patients’ turnover, chronic conditions and acuity and in turn higher and complex care demands. Moreover, staff nurses’ numbers were not adjusted to these challenging conditions. On the contrary, the hospital nursing staff budget proved to be decreased just recently.

“*Our management expects good patient care quality but with a decrease of care personal … not easy (staff nurse interviewee 2).”*

Shorter patients’ turnover gives staff nurses a lot of strain. Many tasks must be done within a short time frame so that staff nurses have to work against the clock.

“*A lot of admissions during the day have an important effect on your workload (staff nurses interviewee 2).”*

The nurse managers addressed that unexpected and unpredicted clinical problems with patients or unexpected admissions were an important reason for the perceived workload.

“*A common thread are unexpected events, it affect us, additionally to our daily activities and require immediately our attention … our usual specialities unpredictability’s and then … and on top of that, Murphy’s law (Nurse manager interviewee 8).”*

Moreover, insufficient communication and lack of vital information exchange between healthcare workers (e.g. physicians, nurses, …) was experienced frustrating. To deal with such situations, staff nurses have to set priorities when they deliver care. Staff nurses level of experience and competencies helped to manage their workload.

“*You have to learn to deal with workload; in the beginning of your career it is very overwhelming (staff nurses interviewee 4).”*

Nurse managers agreed that the amount and the speed of changes in the hospital negatively affected their staff nurses. Current nurse staffing levels at the unit were not feasible to deal with daily care delivery and in addition were not sufficient to integrate changes. Moreover, they asked very tangible to replace absent staff nurses timely.

“*Even experienced teams have difficulties to deal with al these changes. Young staff nurses are more open to changes but we had one young staff nurse, she left us after only 4 months assigned to our unit because of too many changes (Nurse manager interviewee 10).”*

Study participants noticed that work conditions are essential in daily practice to balance workload. Firstly, there were interruptions of care activities such as telephone calls or lack of material and equipment or patients admitted to the unit with care demands other then the usual unit specialty. Secondly, the majority of the study participants referred to the growing problem of paperwork such as patient records (partial paper and electronically), additional registrations such as mandatory governmental and hospital registrations, …

“*A huge obstacle is our patient record system, it is changing all the time and very comprehensive … it is a burden (Nurse manager interviewee 5).*”

In addition, the hospital was involved in a JCI – accreditation process and staff nurses were overwhelmed because of continuous changings in guidelines and procedure with high expectations to comply.

*“Hospital management is trying hard to meet targets within the hospital vision and JCI – requirements but that does not always reflect our daily practice (staff nurses interviewee 2).”*

*“JCI goes too fast within a tight time schedule, staff nurses have not the reasonable time to change their routines properly (Nurse manager interviewee 3).”*

On the other hand, respondents agreed that the JCI – accreditation process stimulates critical thinking about care delivery, quality of care and patient safety.

“*JCI stimulates awareness how things are going in daily practice and how to improve (staff nurse interviewee 7).”*

Innovations and changes through Lean and Productive Ward were more helpful to balance nurses’ workload.

“*Lean is very positive, there is a clear return of investment (staff nurses interviewee 2).”*

Work was better organized, focused more on patient care efficiency and effectiveness through many initiatives supported by staff nurses’ team decisions such as the use of colour codes on material and equipment or bedside nursing handover. Nurse managers respondents agreed that the latter was an example of successful initiatives initiated by Productive Ward with positive outcome on staffs’ workload.

“*I heard many positive comments on bedside nursing handover from staff nurses and patients. An example of a successful changes that impacts workload positively (Nurse manager interviewee 9).”*

However, not all nurse managers were convinced that Productive Ward supports staff nurses’ workload.

“*Maybe little things like to organize better our wound care trolley can be helpful to support staff nurses workload (Nurse manager interviewee 5).”*

#### Interdisciplinary collaboration, communication and teamwork

Collaboration with colleagues and other healthcare workers was supportive and helpful to balance workload. Respondents were clear that interdisciplinary meetings were essential for staff nurses to have the right information about patients and their care demands and in turn for patients too.

“*Often experience and competent physicians have more clear schedules and communication (staff nurse interviewee 9).”*

Nurse managers agreed that communication between colleagues and with physicians was essential and experienced as good and adequate. Besides good collaboration, good and accurate communication was essential within the team as well as with the management level. In order to cope with workload, it is important that staff nurses can express their negative feelings about how things are going.

“*We are a team and together as a team we will deal with workload (staff nurse interviewee 5).”*

*“Sometimes workload is so overwhelming that you have to express your opinion so badly, but meanwhile it is a loss of your energy too (staff nurse interviewee 3).”*

Respondents noticed that there were many ways to express ones’ opinions or to speak up through a short talk or reflection with colleagues or the nurse manager; to grumble and complain and to laugh and weep. However, due to lack of time staff nurses were sometimes bad tempered, snapped to each other and were very defensive; a vicious circle that was recognizable. Workload is unacceptable when teams suffer and team working will be undermined by such a vicious circle.

“*We try to avoid irritations (staff nurse interviewee 6).”*

Remarkable, staff nurses communicate easy about their workload compared to their flaws and mistakes. The latter, often will be kept silence and covered.

“*Often mistakes and flaws will be explained through high workloads and regular swept under the carpet (staff nurses interviewee 5).”*

Communication with the nursing department management level was seen as difficult and with certain barriers. The nurse administration not always listens and responds to staff nurses’ concerns and caused certain tensions.

“*Management communication is often focused to data and numbers (staff nurses interviewee 3).”*

Staff nurse participants reported that striving to meet both patients’ expectations as well as managements’ expectations caused feelings of inadequacy and frustration. Hospital management doesn’t listen always to personnel. Nurse managers expect more visibility of executives and administrators and possibilities to speak with them.

“*A call for help must be answered, I never complain but when I call for help I need someone that listens (Nurse manager interviewee 10).”*

#### Staff nurses personal characteristics and competencies

Study participants agreed that staff nurses must be stress-resistant and must have a strong capacity for self-management in order to cope with daily hassles. Stress-resistance is an important feature of a competent nurse, essential for your patients, yourself and your colleagues, for the hospital as well as society. Study participants agreed also that they experience work stress. Spending enough time to each assigned patients often leads to equal time pressure. At this point, differences between acceptable and unacceptable workload were defined. Acceptable workload is to work hard, to address all your patients care needs and as well as delivering good quality of care.

*“When you work hard focussed on good patient care you can learn every day (staff nurse interviewee 1).”*

Nurse managers confirmed that nurses like to work hard, are eager to learn and that they need certain challenges and pressure. Otherwise it will be boring. Respondents defined unacceptable workload when they could not meet patients care demands resulting in poor quality of care.

*“More then half of our time we experience unacceptable workload (staff nurse interviewee 3).”*

Therefore, the hospital goal of patient centeredness often is neglected and affects nurses in a negative way. Staff nurses will use coping mechanisms such as letting go and being less accessible and approachable.

*“Instead of that we constantly look for and use new coping mechanisms … something must be done … otherwise the hospital will do badly (staff nurse interviewee 3).”*

Respondents reported various impacts of high and prolonged workload such as decreased adequacy and efficacy complains of fatigue, headache and vulnerability for diseases. Mentally, staff nurses complain of failure and impotence, restlessness, frustration, negativity. Some respondents reported that they were often querulous and sad during their work as well as in their personal life; a reason to decrease their working regime. Others reported depressive symptoms and one reported about a colleague ‘s clinical burnout. Some nurse managers saw differences between older and experienced staff nurses and younger more vital staff nurses. The latter were more stressed and chaotic, the first more steady but more reluctant toward changes and innovations. Not all nurse managers were convinced that staff nurses’ clinical burnout was caused by work related factors only. However, all nurse managers understand well and were aware of the risk of high and prolonged workload. One nurse manager was highly affected by a drop out of an experienced colleague through a mental break down. “*She told me that the workload on the unit was the straw that breaks the camel's back (Nurse manager interviewee 6.).*

*“Workload is not the only factor of staff nurses ‘ absenteeism (Nurse managers interviewee 2.).”*

Otherwise stimulating factors were reported such as receiving sufficient recognition from patients and colleagues, interdisciplinary collaboration, a challenging work environment, to love your work and getting social support from colleagues.

“*A good team can balance workload (staff nurses interviewee 1).*”

These stimulating factors prevent intentions to leave the nursing profession. Nurse managers were strongly aware about supporting staff nurses in their daily activities in order to facilitate teamwork and create a good team.

“*I try to motivate staff nurses in every situation also when it is about a decision that I as a nurse manager don’t really support (Nurse managers interviewee 7.).”*

*“ I try to listen and let staff nurses to speak up … an important aspect of our job as a nurse manager (Nurse manager interviewee 2.).”*

*“I support and help staff nurses when we have a lot of work by making telephone calls or arrangements around unplanned patient admissions such as patients from intensive care, … to lower the stress, I try to avoid that my unit will crash (Nurse managers interviewee 10.).”*

Nurse managers addressed that sometimes they cannot anticipate or support staff nurses enough because of high and prolonged workload.

“*Sometimes I have to decide about matters the team don’t like but we have to (Nurse managers interviewee 1.).”*

Nurse managers reported that staff nurses turnover in their unit were low. Some agreed that there were nurses who left their unit or the hospital because of unit workload as well as health problems.

#### Patient centeredness, quality and patient safety

Workload affects not only staff nurses but also patients. Staff nurses were less able to focus on their patients, were less attentive to changes in patient status and clinical signs.

“*Often you are focussed not enough to your patients and overlook important changes; often we overlook early clinical signs (staff nurses interviewee 6*).”

Some respondents reported fear for serious adverse events and in case of adverse events they have cared first for their patients and often have neglected to report safety incidents. Naturally, patient safety aspects such as checking patient identification, fall prevention, prevention of nosocomial infections … are our staff nurses’ daily concern confirmed the nurse managers’ respondents. But they admitted that workload could affect quality of care and patient safety.

“*Our staff nurses have to work fast and are afraid to make mistakes, … sometimes they have the feeling that they deliver unsafe patient care … (Nurse manager interviewee 3.).*

To often, due to high work demands staff nurses have to make choices. Instead, they will provide total care.

“*I admit to evaluate patients’ pain scores regularly is important but I prefer that staff nurses administer pain medication 4 times a day (Nurse manager interviewee 3.).”*

Study participants agreed that the main impact of workload is the lack of social interaction with patients.

“*You have to set priorities and the first thing you loose are the opportunities for social interaction with patients (staff nurses interviewee 6).”*

Patient communication and information about diagnostics and treatment were briefer and patients’ questions and worries were more neglected.

*“Quality of care equals listen to patients (staff nurses interviewee 8).”*

Nurse managers addressed that a lot of staff nurses’ frustration originated from their inability to meet patients’ need. Staff nurses consider this failure.

“*Lack of time for patients’ mental and emotional well-being is a source of staff nurses’ frustration (Nurse managers interviewee 6.).”*

Remarkable, a lot of patients admire the nursing workforce. Patients perceive differences in workload and often accept the consequences.

“*As a nurse you have the impression that you fall short more then patients’ impression of our shortcoming (staff nurses interviewee 9).”*

### Models explaining and interpreting using qualitative study findings

Study participants addressed a bundle of factors that influenced workload. These factors described how daily practice was organized and certain conditions were in place (*nurse management at the unit level*) largely determined by management decisions and policy (*hospital management & organizational support*). In turn, workload clearly was a risk factor for staff nurses’ symptoms such as fatigue, headaches and vulnerability for diseases (*emotional exhaustion*), for negative feelings such as frustration and negativism and behaviours such as letting go, being less accessible and approachable (*depersonalisation*) as well as thoughts of failure and inefficacies (*personal accomplishment*) to patients needs and demands (*quality of care items*). Good interdisciplinary collaboration and communication (*nurse – physician relations*) that supported nursing practice (*decision latitude*) as well as supportive collaboration between colleagues such as good teamwork, opportunities to speak up and express opinions (*social capital*) were protective factors to balance workload; to deal with stressful work conditions, to be engaged for patients total patient care (*vigor and dedication*) and to stay in the nursing profession (*job outcome items: intention to stay in the profession*). Study participants expressed their concerns about the impact of high and prolonged workload on quality and patient safety (*quality of care items)* through nurses’ mistakes, which often were not reported. Participants were concerned that they might overlook relevant patients’ vital and other clinical signs as well as neglect patients’ mental and emotional needs. Both staff nurses and nurse managers reported staff nurses’ feelings of sadness and querulousness (*job outcome items: satisfaction with the current job*). Predictions of favourable hospital management & organizational support as well as nurse management at the unit on workload and study outcomes were confirmed: study participants reported supportive work conditions through successful innovations that engaged staff and improved patients’ care and well-being. Moreover, nurse unit managers showed that they have a pivotal position between management decisions and daily practice and work conditions supporting and protecting their team and teamwork.

## Discussion

In the quantitative study the two retested models with burnout and engagement as mediating outcome variables were largely confirmed with a convenient study sample in two acute care university hospitals. Our study results are in line with previous studies about hospital Magnet status showing the relevance of hospital-level and unit-specific strategies to achieve an excellent nursing practice environment [14, 22, 24]. Moreover, in additional analysis of models the qualitative study findings confirmed associations described in both quantitative studied models. Study participants explained the important impact of management and policy decisions on their daily practice as well as the role of their peers and nurse manager and good interdisciplinary relationship with physicians. Laschinger et al. [29] showed that nurse managers’ authentic leadership behaviour such as self-awareness and transparency, moral-ethical behaviour and supporting balanced processes plays an important role in creating positive working conditions. In addition, this behaviour strengthening new nurses’ confidence that helps them to cope with increased job demands and protect them from feelings of burnout and poor mental health. The models as well as what staff nurses’ and nurse managers’ expressed in the qualitative study identified and confirmed risk factors as well as protective factors related to favourable job outcomes and nurses’ assessed quality of care. Social capital and decision latitude are nurse work characteristics that are strongly predicted by nurse management at the unit level. In turn, social capital has a protective and stimulating impact on emotional exhaustion and vigor. Furthermore, decision latitude has a stimulating impact on personal accomplishment and dedication. In an empowered work environment nurses have access to relevant information, opportunities for learning and personal development and supportive relationships with peers, supervisors and interdisciplinary to achieve their goals. Moreover, professional discretion and visibility, strong commitment, engagement, work effectiveness and quality of care were identified [28, 52, 53, 57]. Instead, workload showed to be a relevant risk factor predicted by hospital management and organizational support with a highly negative impact on emotional exhaustion and vigor as well as on both outcome variables. The qualitative study revealed clearly the differences between acceptable and unacceptable workload as the capacity nurses have to sufficiently meet patients’ physical as well as emotional needs. In addition, when staff nurses were able to consider patients’ status and clinical signs timely providing quality and patient safety that also resulted in acceptable workload perception. High and prolonged workloads were related to nurses’ decreased adequacy and efficacy, complains of fatigue, headache and vulnerability for diseases as well as affects nurses’ feelings of frustration, negativity and sadness. These feelings could affect not only the individual nurse but also the whole team [44, 50]. A study investigating nursing performance under high workload revealed that certain mechanisms such as selection, optimization and compensation strategies (SOC model) support nurses’ individual decision-making and ability to perform well [9]. The SOC model implicates that nurses use their individual resources more efficiently and adaptively by setting priorities and focus on fewer but most relevant goals, pursue these goals in an optimized way and flexibly apply compensatory means [10]. More research on staff nurses’ cognitive and physical workloads and work demands [23] within an supportive and empowered psychosocial work environment will offer better insights in achieving a healthy nurse workforce and excellent quality and safety of care. However, personality characteristics in nurses vulnerable to develop burnout are identified and require sufficient and appropriate attention [19].

### Limitations

Certain limitations of the study are recognized. Firstly, although retested and confirmed, the models were based on a cross-sectional study design and should be interpret with caution. A longitudinal study design could confirm and/or extent our study results. Secondly, the qualitative study was performed independently of the model retesting and confirmation and gave additional insights about the studied variables and pathways between variables through additional model analysis. However, the study was conducted with staff nurses and nurse managers of medical and surgical wards of one study hospital. Other wards and the second hospital were not involved. Future qualitative research with other wards such as obstetric, geriatric and/or intensive care units or services such as operation theatre could confirm and extent study results. Thirdly, both study methods were based on nurses’ perceptions and experiences. Additional study method involving objective nurse and patient related variables could extent confirmation of our study results. Finally, replication in different socio-economic conditions is necessary to support generalizability.

## Conclusion

This mixed method study based on an explanatory sequential study design provides a deep understanding of various associations and impacts on studied outcome variables. Risk factors and protective factors were identified through the retested and confirmed models and corresponded closely what study participants revealed. Besides the more *softer* work characteristics such as decision latitude and social capital and team cohesion more insight and knowledge of the *hard* work characteristic workload is essential.

## Abbreviations

AMOS: Analysis of Moment Structures
ANCC: American Nurses Credentialing Center
CFI: Comparative Fit Index
IFI: Incremental Fit Index
JCI: Joint Commission International
MBI-HSS: Maslach Burnout Inventory-Human Service Survey
NHS: National Health system
NVivo: Qualitative data analysis computer software
NWI-R: Revised Nursing Work Index
RMSEA: Root Mean Square Error of Approximation
SPSS: Statistical Package for the Social Science
UWES: Utrecht Work Engagement Scale
